# Dynamic Succession of Soil Bacterial Community during Continuous Cropping of Peanut (*Arachis hypogaea* L.)

**DOI:** 10.1371/journal.pone.0101355

**Published:** 2014-07-10

**Authors:** Mingna Chen, Xiao Li, Qingli Yang, Xiaoyuan Chi, Lijuan Pan, Na Chen, Zhen Yang, Tong Wang, Mian Wang, Shanlin Yu

**Affiliations:** Shandong peanut research institute, Qingdao, China; Graz University of Technology (TU Graz), Austria

## Abstract

Plant health and soil fertility are affected by plant–microbial interactions in soils. Peanut is an important oil crop worldwide and shows considerable adaptability, but growth and yield are negatively affected by continuous cropping. In this study, 16S rRNA gene clone library analyses were used to study the succession of soil bacterial communities under continuous peanut cultivation. Six libraries were constructed for peanut over three continuous cropping cycles and during its seedling and pod-maturing growth stages. Cluster analyses indicated that soil bacterial assemblages obtained from the same peanut cropping cycle were similar, regardless of growth period. The diversity of bacterial sequences identified in each growth stage library of the three peanut cropping cycles was high and these sequences were affiliated with 21 bacterial groups. Eight phyla: *Acidobacteria, Actinobacteria*, *Bacteroidetes*, *Chloroflexi*, *Gemmatimonadetes*, *Planctomycetes*, *Proteobacteria* and *Verrucomicrobia* were dominant. The related bacterial phylotypes dynamic changed during continuous cropping progress of peanut. This study demonstrated that the bacterial populations especially the beneficial populations were positively selected. The simplification of the beneficial microbial communities such as the phylotypes of *Alteromonadales*, *Burkholderiales, Flavobacteriales*, *Pseudomonadales, Rhizobiales* and *Rhodospirillales* could be important factors contributing to the decline in peanut yield under continuous cropping. The microbial phylotypes that did not successively changed with continuous cropping, such as populations related to *Rhizobiales* and *Rhodospirillales*, could potentially resist stress due to continuous cropping and deserve attention. In addition, some phylotypes, such as *Acidobacteriales*, *Chromatiales* and *Gemmatimonadales*, showed a contrary tendency, their abundance or diversity increased with continuous peanut cropping progress. Some bacterial phylotypes including *Acidobacteriales, Burkholderiales, Bdellovibrionales,* and so on, also were affected by plant age.

## Introduction

The bacterial populations in the root related environment contribute to plant health by mediating nutrient acquisition, by causing soil borne diseases or by controlling the growth or activity of plant pathogens [1], [2], [3]. The interactions between soil, bacteria and plants in the root related environment determine soil fertility and sustainability and plant quality [3], [4]. The structural and functional diversity of soil bacterial communities are influenced by several biotic and abiotic factors, such as climate and season, soil type and structure, treatment or management of the soil, plant species and development stage [5], [6], [7], [8], [9], [10]. In agriculture, soil type, crop selection and cropping sequence have been shown to be the key determining factors on soil microbial communities [6], [11], [12]. On the other hand, microbial community characteristics have been shown to be efficient bio-indicators of soil condition and land management success due to their quick response to environmental changes, although soil productivity and sustainability depends on a complex of physical, chemical and biological properties [13], [14]. Because of the interactions among bacteria, soil and plants, bacterial community succession has been shown to be a potential indicator of environmental change [13], [14], [15] and active management of soil bacterial communities, such as the manipulation of microbial populations, could be a promising approach to improving soil conditions, developing natural suppression of soil borne diseases and improving crop productivity [4], [16]. However, the specific populations, characteristics and relationships between plants and soil bacterial communities are known relatively little.

Peanut, an important oil and economic crop worldwide, is very adaptable to climatic conditions and grows in many different environments between latitudes 40°N and 40°S [17]. However, its productivity is negatively affected by continuous cropping. The yield of other main world crops, such as wheat, rice, corn and soybean, also decline under continuous cropping [18], [19], [20]. It has been reported that the negative influence of continuous cropping on soil productivity has a significant effect on soil microbial biomass and community structure [21], [22]. Previously, we investigated the succession characteristics of soil eukaryotic microorganisms under continuous peanut cropping and found that soil eukaryotic microorganisms showed significant dynamic changes during continuous cropping, especially with regards to the pathogenic and beneficial fungi selected [23]. However, there is no comprehensive knowledge of specific soil bacterial inhabitants, characteristics, succession pattern and how soil bacteria interact with continuous peanut cropping.

This investigation is an attempt to study the soil bacterial community characteristics of peanut and bacterial succession under continuous peanut cultivation by comparing the soil bacterial community structure when peanut was grown over three continuous cropping cycles and its representative growth stages. This was achieved by using cultivation-independent 16S rRNA gene clone library analyses. The aims of this study were to determine the phylogenetic affiliations of the most common and predominant populations of soil bacteria at different peanut growth stages under a continuous cropping regime as well as identify the diversity and succession patterns of soil bacteria during continuous cropping progress.

## Materials and Methods

### Ethics statement

No specific permits were required for the described field studies. No specific permissions were required for these locations and activities. The location is not privately-owned or protected in any way and the field studies did not involve endangered or protected species.

### Management description and soil sampling

The study was conducted between 2008 and 2010 at Shandong Peanut Research Institute in Qingdao, China (36.10°N, 120.41°E). Peanuts were planted in May each year and harvested in October. Pot culture experiments were undertaken and fourty Huayu 25 seedlings (a pot a seedling) were planted, each year. The soil consisted of 28.5% clay, 41% loam, and 30.5% sand, with no known history of peanut cropping. In order to reduce interference from external factors on the soil bacterial community: The same peanut variety was employed during all the cropping cycles; two drought sheds were built, the plants were irrigated with sterile water and no fertilizer was applied during the growing seasons and fallow periods; Samples were collected at the same time of the three planting cycles, and at 5–10 cm soil depths. Two different peanut growth stages in the three cycles were selected: the seedling stage (twenty days after planting), and the pod-maturing stage (harvest time, about 129 days after planting). The soils were sampled using a soil probe (1.5 cm diameter) at 5–10 cm soil depths and five replicate test plants were used for each stage. The samples were stored at −80°C and were used for bacterial community analysis.

### Genomic DNA extraction and 16S rRNA gene library construction

DNA was extracted from the pooled material for each sample with Ultra-Clean soil DNA kits (MoBio, USA). Bacteria-specific primers, 27F and 1387R primers [24], were used for 16S rRNA gene amplification. The reaction was performed for 25 cycles using the ‘Easy-A High-Fidelity’ PCR master mix from Stratagene (USA). Products from six PCRs were pooled, purified, using a Gel Extraction kit (Omega, USA), and ligated into pMD19-T simple vectors (Takara, Japan). The hybrid vectors were cloned into *Escherichia coli* TOP10. Clone libraries were constructed by random selection of approximately 100 white colonies from a single plate for each library. All clone libraries were named on the basis of the crop cycle and the plant growth stage of soil sampling. S1 and M1 were libraries constructed respectively for the seedling stage and pod-maturing stage in the first crop cycle; S2 and M2 were libraries constructed respectively for the two stages in the second cycle and S3 and M3 were libraries constructed respectively for the two stages in the third cycle.

### 16S rRNA gene sequencing and data analysis

Cloned 16S rRNA gene fragments were re-amplified by using primers RV-M (5′-GAGCGGATAACAATTTCACACAGG-3′) and M13-D (5′-AGGGTTTTCCCAGTCACGACG-3′) that flanked the insertion site of the cloning vector. Two primers, V-27 and V-1387 [25] spaned both the pMD19-T simple cloning vector RV-M annealing end and one of the 16S rRNA gene insert ends. The primers were used separately with primer M13-D for PCR determination of the orientations of the inserts. Primer M13-D or RV-M was chosen based on insert orientation and all positive clones were sequenced to improve data accuracy and integrity.

Operational taxonomic units (OTUs) in each library were defined by using the Mothur program at the 3% distance level [26]. Chimeric sequences were identified using Greengenes, a chimera-checked 16S rRNA gene database [27]. Sampling efficiency and the sequence phylotype diversity of the libraries were analyzed using the rarefaction curve method and the aRarefactWin program [25]. Similarities between the soil bacterial assemblages at different peanut growth stages during different crop cycles were established by performing cluster analyses using the software package SPSS 11.5 (SPSS, United States). The frequency (*F*) for the OTUs was calculated as follows: *F* = (*m*/*N*)×100, where *m* is the clone number of an OTU in a library and *N* is the total number of clones in the same library. Log-transformed frequency data were used to minimize the influence of preferential PCR amplification [25], [28]. The relative abundance and occurrence of the bacterial orders or phylotypes detected in the six 16S rRNA gene libraries were visualized as a heatmap using Genesis program 1.7. Sample clustering and bacterial phylotype clustering were analyzed by using complete linkage clustering method.

The nearest phylogenetic neighbor sequence for each OTU sequence was determined in the GenBank database using the BLASTN program [29]. Sequences were aligned using ClustalX v.1.81.9 [30]. Phylogenetic trees were constructed using the DNADIST and NEIGHBOR programs in the PHYLIP package (version 3.65c) [31] and bootstrap support was evaluated using 100 replicates.

### Nucleotide sequence accession numbers

The 16S rRNA gene sequences determined have been deposited in the GenBank database under accession numbers KF182746–KF183313.

## Results

### Statistical and comparative analyses of the six libraries

Six 16S rRNA gene libraries were constructed over three continuous cropping cycles and over two peanut growth stages for each cycle. A total of 618 clones were screened and 576 of them contained a proper insert. A total of 351 OTUs were identified, eight of which (eight clones) were probably chimeras, based on the chimera-check results. Rarefaction analysis indicated the high OTU phylotype diversity of the soil bacteria (Figure S1).

**Figure 1:**
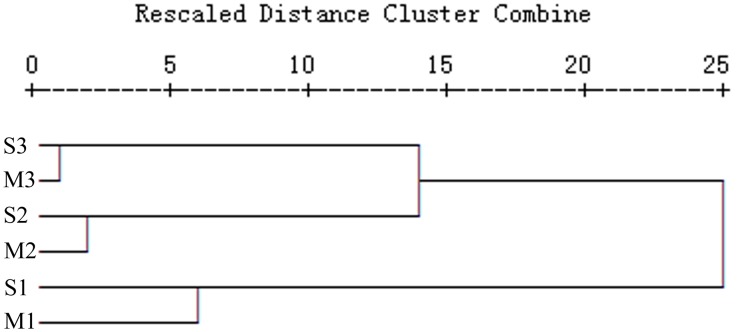
Dendrogram based on a hierarchical clustering analysis of the 16S rRNA gene clone libraries, constructed using the squared Euclidean distance similarity and Ward linkage procedures.

**Figure 2:**
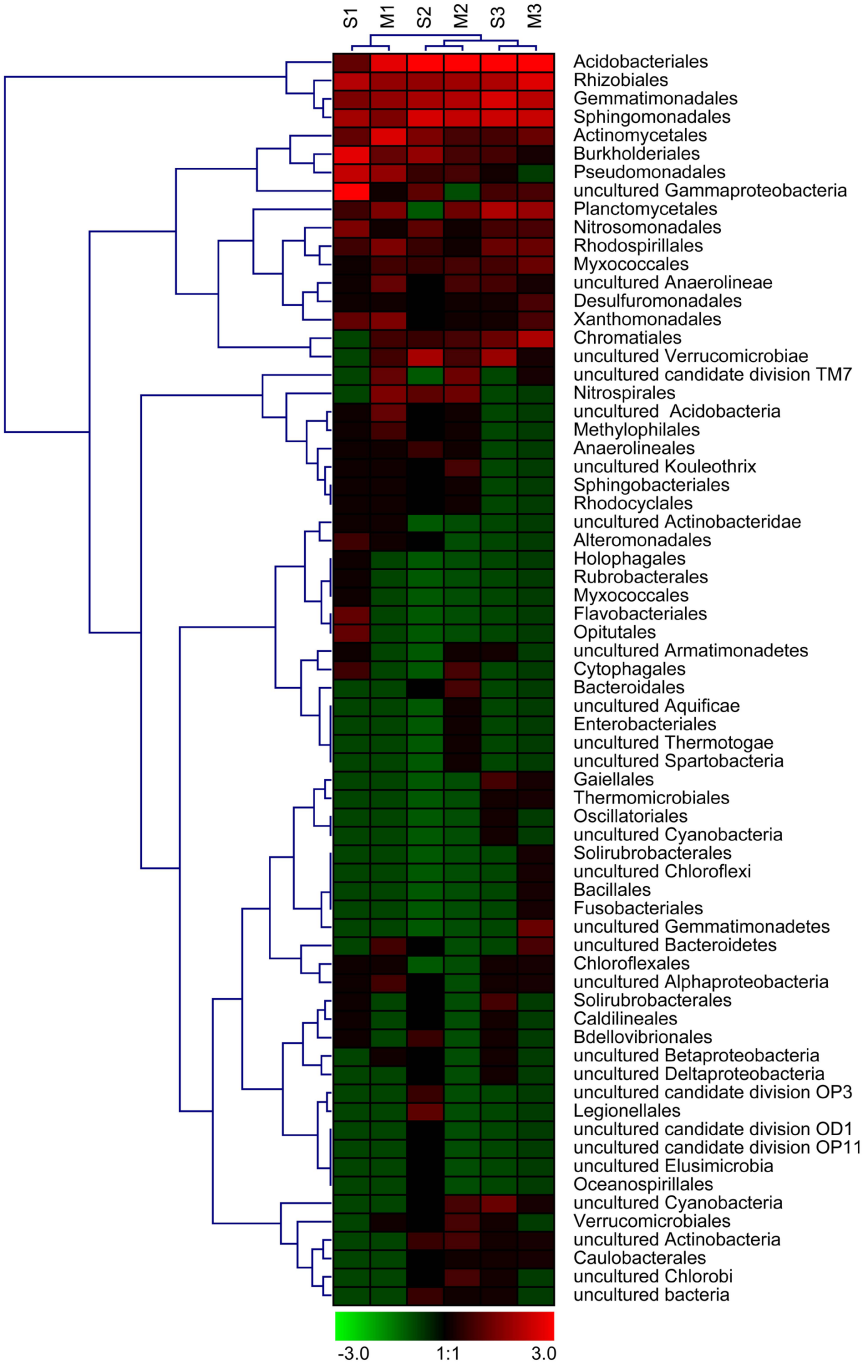
Heatmap analyses of the bacterial orders or phylotypes detected in the six 16S rRNA gene libraries. The color code indicates relative abundance, ranging from green (low abundance) to black to red (high abundance).

The OTU number ranged from 69 to 82 in the six libraries. The clone libraries obtained from the same cycle, but from different growth stages, typically shared 15 to 17 common OTUs, accounting for 20.0 to 23.2% of the OTUs in each library (Table 1). The dynamics of the bacterial community structure were further analyzed by performing cluster analyses. The hierarchical (Figure 1) and k-means (Figure S2) as well as heatmap sample (Figure 2) clustering results were consistent and showed that the libraries obtained from different growing stages, but from the same cropping cycle, were grouped together. The results demonstrated that the soil bacterial populations changed when peanut was grown continually, regardless of growth stage.

**Table 1:** Analysis of the clone libraries constructed over three continuous cropping cycles and representative growing stages of peanut.

Library	No. of clones	No. of OTUs	No. of uniqueOTUs	No. of OTUsshared bylibraries fromthe samecropping cycle	% shared OTUs
S1	99	69	58	16	23.2
M1	92	80	69		20.0
S2	103	82	67	17	20.7
M2	90	79	71		21.5
S3	90	75	64	15	20.0
M3	94	75	62		20.0

### 16S rRNA gene sequence analyses

**Figure 3:**
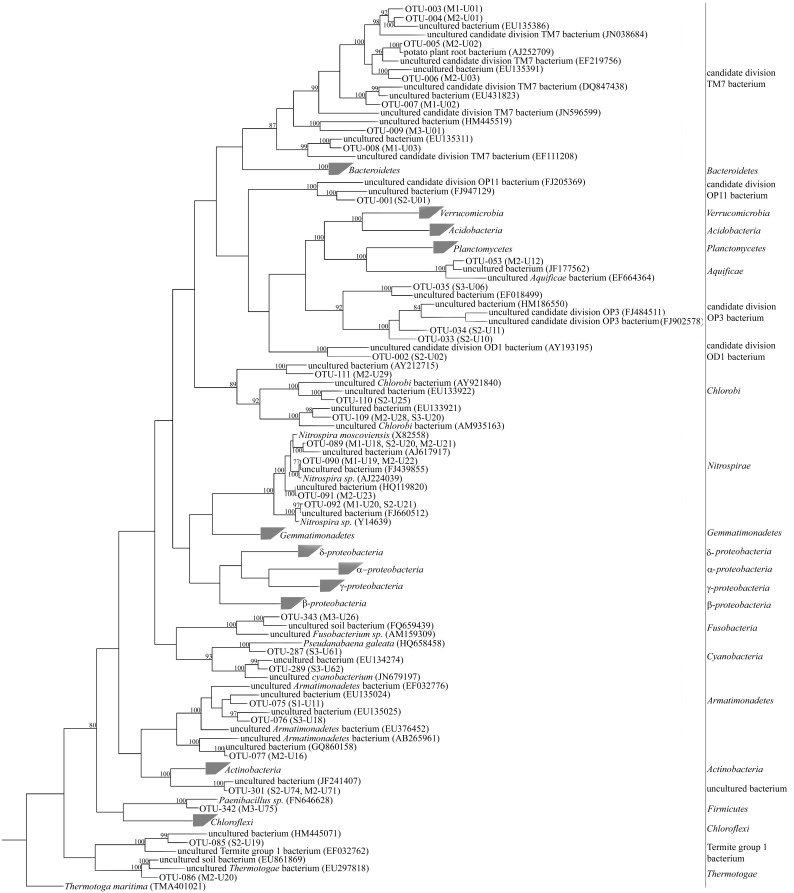
Phylogenetic tree of the bacterial sequences recovered from the six libraries, constructed using the neighbor-joining method with the Kimura two-parameter model for nucleotide change. The predominant groups which were just denoted the phylogenetic positions in this tree, presented in other separate phylogenetic trees. The libraries OTUs occurred were labeled. Scale bar, 0.1 substitutions per nucleotide position. Bootstrap values (100 replicates) above 70% are indicated at the nodes. The tree was rooted using the sequence related to *Thermotoga maritima* (TMA401021).

**Figure 4:**
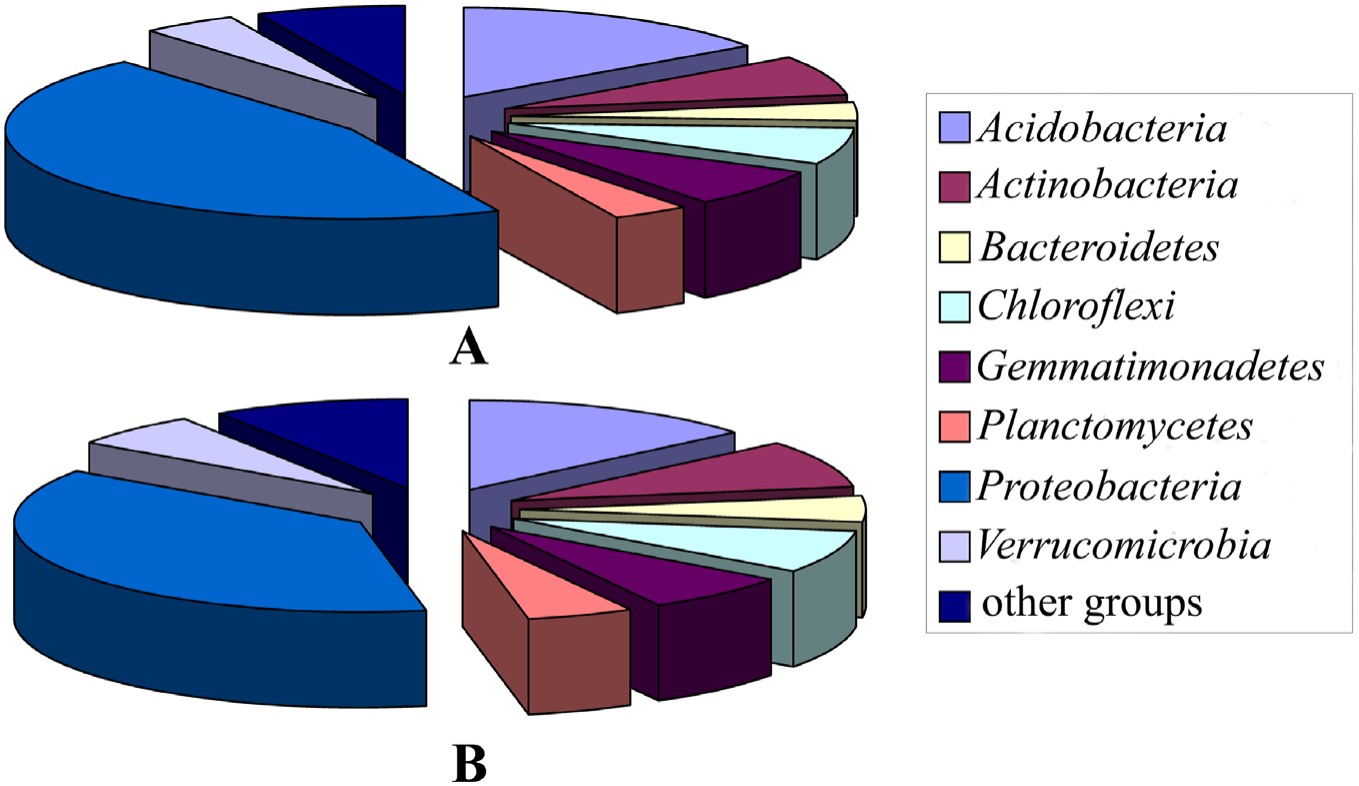
The abundance and diversity analyses of the bacterial groups identified in the six libraries. A shows the clone percentage of these groups and B shows the OTU percentage.

All the 343 valid OTUs were used for sequence analysis, most (74.9%) of which showed high levels of similarity (≥97% identity) with known sequences in the GenBank database and 12.9% were moderately related (≤94% identity) to known sequences. Phylogenetic analysis showed that 342 OTUs were affiliated with the domain, *Bacteria*, and the remaining OTU was related to the chloroplast 16S rRNA gene from *Daviesia angulata*. There were 21 groups related to *Bacteria* identified in the libraries (Figure 3). Eight phyla: *Acidobacteria, Actinobacteria*, *Bacteroidetes*, *Chloroflexi*, *Gemmatimonadetes*, *Planctomycetes*, *Proteobacteria* and *Verrucomicrobia* were predominant, accounting for 93.0% of the 568 clones and 91.0% of the 343 valid OTUs (Figure 4). The phylum, *Proteobacteria*, dominated in all the libraries, accounting for 45.6% of the 568 clones, and showed the greatest diversity, accounting for 37.9% of the 343 OTUs. The phylum, *Acidobacteria* was relatively abundant, accounting for 15.3% of the obtained valid clones and was highly diverse, accounting for 14.0% of the OTUs analyzed. The other six phyla sequences accounted for 3.4 to 7.2% of the obtained clones and 4.7 to 8.5% of the analyzed OTUs (Figure 4). The bacterial orders or phylotypes affiliated with these phyla showed dynamic succession with continuous cropping of peanut (Figure 2).

#### *1) Proteobacteria* sequences

One hundred and thirty OTUs (45.6% of the clones) were related to *Proteobacteria*, which predominated in all the libraries and showed the greatest diversity. Four classes: α-*proteobacteria*, β-*proteobacteria*, δ*-proteobacteria* and γ-*proteobacteria*, were affiliated with the *Proteobacteria* phylum (Figure 3). The α-*proteobacteria* sequences were most abundant and diverse, accounting for 40.9% of the *Proteobacteria* clones and for 39.2% of the corresponding OTUs. The γ-*proteobacteria* and β-*proteobacteria* sequences were relatively abundant, accounting for 30.5 and 18.9% of the *Proteobacteria* clones, respectively, and were relatively diverse, accounting for 23.8 and 20.7% of the corresponding OTUs, respectively. The δ*-proteobacteria* sequences accounted for 9.7% of the *Proteobacteria* clones and for 16.2% of the corresponding OTUs. All four class sequences showed considerable dynamic population changes.

**Figure 5:**
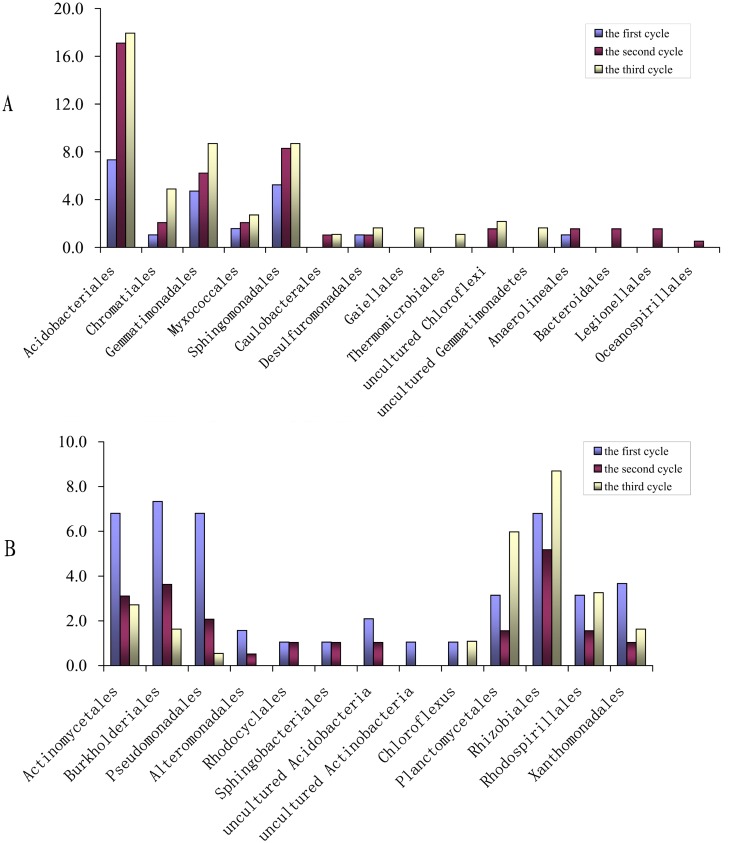
The clone abundance analyses of the bacterial orders or phylotypes which showed succession changes in populations with continuous cropping cycles. (The clone abundance was calculated as: *m*1/*M*×100, where *m*1 is the number of related clones detected in the libraries for the same cropping cycle and *M* is the total number of clones in the same libraries.)

The α-*proteobacteria* sequences were abundant and diverse in all clone libraries and the clone abundance accounted for 16.8, 16.6 and 22.8%, respectively, of the first, second and third cycle library clones. Five orders or subdivisions were identified as being related to α-*proteobacteria*, including the orders of *Caulobacterales*, *Rhizobiales*, *Rhodospirillales*, *Sphingomonadales*, and an uncultured α-*proteobacteria* subdivision (Figure S3). These subdivisions showed complex dynamic changes with continuous peanut cropping (Figure 2, Figure 5). The *Rhizobiales* and *Sphingomonadales* sequences were the most abundant α-*proteobacteria* members in the libraries, accounting for 34.9 and 39.6%, respectively, of the 106 α-*proteobacteria* clones. The sequences affiliated with *Rhizobiales* were diverse, accounting for 47.1% of the 51 α-*proteobacteria* OTUs. Their abundance slightly decreased and then increased during continuous cropping, accounting for 7.1, 5.4, 4.9, 5.6, 6.7 and 10.6%, of the clones identified in the six libraries, respectively. Their clone diversity showed a similar tendency, accounting for 8.7, 6.3, 3.7, 6.3, 8.0 and 9.3%, respectively, of the OTUs identified in the six libraries. Eleven OTUs were related to *Sphingomonadales* and the clone abundance accounted for 5.2, 8.3 and 8.7%, respectively, of the clones obtained in the three cycle libraries. Seven OTUs were related to *Rhodospirillales* and accounted for 3.1, 1.6 and 3.3%, respectively, of the clones obtained in the three cycle libraries. Only two OTUs were related to the *Caulobacterales* order and these sequences appeared in all the second and third cycle libraries, but were not found in any first cycle libraries. The OTU related to *Myxococcales* was only identified in library S1.

**Figure 6:**
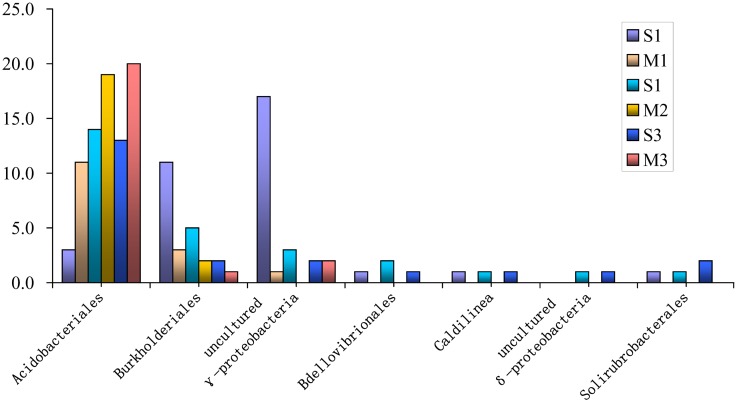
The clone abundance analyses of the bacterial orders or phylotypes which showed succession changes in populations with peanut growth stage. (The clone abundance was calculated as: (*n*1/*N*×100, where *n*1 is the number of related clones detected in a library and *N* is the total number of clones in the same library.)

Thirty-one OTUs were affiliated with γ-*proteobacteria* and the related clones accounted for 22.5% of the clones in the first cycle libraries, but only 9.8, and 9.2% of the clones in the second and third cycle libraries, respectively. These sequences were highly diverse and were affiliated with eight orders or subdivisions: *Alteromonadales*, *Chromatiales*, *Enterobacteriales*, *Legionellales*, *Oceanospirillales*, *Pseudomonadales*, *Xanthomonadales* and an uncultured γ-*proteobacteria* subdivision (Figure S4). These subdivision sequences showed dynamic population changes with continuous cropping (Figure 2, Figure 5). The clones affiliated with *Chromatiales*, *Pseudomonadales*, *Xanthomonadales* and the uncultured γ-*proteobacteria* subdivision were relatively abundant and common in the analyzed libraries, accounting for 19.0, 22.8, 15.2 and 31.6%, respectively, of the 79 γ-*proteobacteria* clones. Six OTUs were affiliated with *Chromatiales* and appeared from the pod-maturing stage of the first cycle. Their abundance increased with continuous cropping and accounted for 1.0, 2.1 and 4.9% of the clones identified in the three cycle libraries, respectively. The sequences affiliated with *Pseudomonadales* were seen in five libraries, but not in library M3. The clone abundance and diversity decreased with continuous cropping and accounted for 6.8, 2.1 and 0.5%, respectively, of the clones obtained in the three cycle libraries. The OTU number was 4, 3 and 1 in the three cycle libraries, respectively. Seven OTUs were related to *Xanthomonadales* and were common in all six libraries, accounting for 3.7, 1.0 and 1.6%, respectively, of the clones obtained in the three cycle libraries. The uncultured γ-*proteobacteria* sequences in the seedling stage library of the first cycle were most abundant, accounting for 17.2% of the clones identified in library S1 and decreased considerably from the pod-maturing stage of the first cycle, accounting for no more than 3% of the corresponding clones in the later five libraries. In addition, the clone abundance in the seedling stage library was higher than in the pod-maturing stage library for the same cycle (Figure 6) and the clone diversity showed a similar tendency, accounting for 4.4, 1.3, 3.6, 0, 2.7 and 1.3% respectively of the OTUs identified in libraries: S1, M1, S2, M2, S3 and M3. Only three OTUs were affiliated with *Alteromonadales* and were seen only in the S1, M1 and S2 libraries, but did not occur in the other three libraries during the later stages. Sequences related with the other three orders were rare and only seen in the second cycle libraries.

Twenty-seven OTUs were affiliated with β-*proteobacteria* and the clone abundance decreased significantly with continuous cropping, accounting for 13.1, 8.3 and 4.3% of the clones detected in the three cycle libraries, respectively. These sequences were associated with orders or subdivisions of *Burkholderiales*, *Methylophilales*, *Nitrosomonadales*, *Rhodocyclales* and an uncultured β*-proteobacteria* subdivision with 96–98% similarity (Figure S5). The sequences related to *Burkholderiale,* which was the dominant β*-proteobacteria* phylotype in the libraries, accounted for 55.6% of the 27 OTUs and 49.0% of the 49 related clones. The clone abundance decreased significantly in the same growth stage libraries with continuous cropping and accounted for 11.1, 4.9 and 2.2% of the clones detected in the seedling stage libraries of the three cycles, respectively, and 3.3, 2.2 and 1.1% of the clones in the three pod-maturing stage libraries, respectively (Figure 5, Figure 6). The clone diversity showed a similar tendency, accounting for 10.1, 4.9 and 2.7% of the OTUs identified in the three seedling stage libraries, respectively, and 3.8, 2.5, and 1.3% of the OTUs identified in the three pod-maturing stage libraries, respectively. The analyses also showed that the clone abundance and diversity in the seedling stage libraries were higher than in the pod-maturing stage libraries of the same cycle. The Sequence related to the orders of *Methylophilales* and *Rhodocyclales* occurred in all the first two cycle libraries, but were not seen in any third cycle libraries (Figure 5). The *Nitrosomonadales* and uncultured β*-proteobacteria* sequences showed no significant changes over the three cycle libraries.

The abundance of δ*-proteobacteria* sequences was low and moderately increased over the three cycle libraries, accounting for 3.1, 4.7 and 5.4% of the corresponding library clones, respectively. These sequences were affiliated with the orders: *Bdellovibrionales*, *Desulfuromonadales*, *Myxococcales* and an uncultured δ*-proteobacteria* subdivision (Figure S6). The clone diversity and abundance of the *Desulfuromonadales* and *Myxococcales* sequences showed a slightly increasing tendency during continuous cropping (Figure 5). The OTU number for the *Desulfuromonadales* clones was 1, 2 and 3 in the three cycle libraries, respectively, and the related clones accounted for 1.0, 1.0 and 1.6%, respectively, of the clones obtained in the three cycle libraries. The OTU number of *Myxococcales* clones respectively was 3, 4 and 5 and the related clones accouted for 1.6, 2.1 and 2.7% of the clones in the three cycles, respectively. Sequences affiliated with *Bdellovibrionales* (3 OTUs) occurred in all three cycle libraries, but only occurred in the seedling stage libraries. The uncultured δ*-proteobacteria* sequences (2 OTUs) were found in the second and third cycle libraries and only occurred in the seedling stage libraries (Figure 6).

#### *2) Acidobacteria* sequences

Forty-eight OTUs (15.3% of the total clones) were affiliated with the *Acidobacteria* phylum. Two classes of *Acidobacteria* and *Holophagae* were identified as being related to *Acidobacteria* in the analyzed libraries. Forty two OTUs were affiliated with the *Acidobacteria* class, which was the predominant phylotype of the *Acidobacteria* phylum and accounted for 92.0% of the corresponding clones. Only one OTU (1 clone) was related to *Holophagae*; the remaining five OTUs were related to uncultured *Acidobacteria* (Figure S7). The three subgroups all showed dynamic changes with continuous cropping (Figure 5). All *Acidobacteria* class sequences were affiliated with the *Acidobacteriales* orde and the abundance significantly increased in the same growth stage library of the second cycle, but there were no significant differences between the same growth stage libraries of the second and third cycles. The related clones accounted for 3.0, 13.6 and 14.4%, respectively, of the clones identified in the seeding stage libraries of each cycle and for 12.0, 21.1 and 21.3% of the clones identified in the pod-maturing stage libraries, respectively (Figure 6). Clone diversity also showed a similar tendency. There were 3, 8 and 8 OTUs identified in the seeding stage libraries of each cycle, respectively, and there were 8, 14 and 17 OTUs identified in the pod-maturing stage libraries, respectively. The analysis also indicated that clone abundance and diversity both increased during the pod-maturing stage compared to the seedling stage of the same cropping cycle. The *Holophagae* sequence, which was affiliated with uncultured *Holophaga sp*. with 96% similarity, was only seen in library S1. The five OTUs related to uncultured *Acidobacteria* occurred in all the four first and second cycle libraries, but not in any third cycle libraries.

#### 3) Sequences of other relatively abundant groups

The *Actinobacteria* sequences were relatively abundant and diverse, accounting for 7.2% of the total clones and 8.5% of the analyzed OTUs. These sequences were affiliated with two classes of *Actinobacteridae*, *Rubrobacteridae* and uncultured *Actinobacteria* (Figure S8). Phylotypes affiliated with these three classes or subgroups showed dynamic changes over the three cycle libraries (Figure 5). The sequences related to *Actinobacteridae* were most abundant and diverse, accounting for 63.0% of the *Actinobacteria* clones and 69.0% of the corresponding OTUs. The *Actinomycetales* orders and an uncultured *Actinobacteridae* subdivision were identified in libraries affiliated with the *Actinobacteridae* class (Figure S8). The *Actinomycetales* clone abundance decreased in the second cycle libraries, but there were no significant differences between the second and third cycle libraries, which accounted for 7.9, 3.1 and 3.3%, respectively, of the clones in the three cycle libraries. Clone diversity showed a similar tendency and the OTU numbers were 10, 5 and 5, respectively, in the three cycle libraries. The OTU related to uncultured *Actinobacteridae* only occurred in the two first cycle libraries, S1 and M1. Three orders: *Rubrobacterales*, *Solirubrobacterales* and *Gaiellales* were identified as being related to the *Rubrobacteridae* class (Figure S8). Four OTUs were affiliated with the *Solirubrobacterales* order and were seen in all three seedling stage libraries and the pod-maturing stage library of the third cropping cycle, but did not occur in the earlier two pod-maturing stage libraries (Figure 6). The OTU related to *Rubrobacterales* only occurred in library S1, but the OTU related to *Gaiellales* only occurred in the two third cycle libraries, S3 and M3. Three OTUs (14.6% of the *Actinobacteria* clones) were affiliated with uncultured *Actinobacteria* and these sequences were seen in all the four second and third cycle libraries, but did not occur in any first cycle libraries.

The sequences related to the *Chloroflexi* phylum accounted for 6.5% of the 568 clones and for 7.9% of the 343 valid OTUs. There were 19 OTUs being affiliated with classes of *Anaerolineae*, *Caldilineae*, *Thermomicrobia* and the remaining 8 OTUs were related to uncultured *Chloroflexi* sequences and the genus, *Kouleothrix*, which has not been fully described in the GenBank database (Figure S9). Ten OTUs (45.5% of the *Chloroflexi* clones) were affiliated with the *Anaerolineae* class and these sequences were related to *Anaerolineales* and uncultured *Anaerolineae* sequences. The *Anaerolineales* sequences were only seen in the four first and second cycle libraries (Figure 5), and the uncultured *Anaerolineae* sequences did not show any dynamic tendencies over the three cycle libraries. Six OTUs (seven clones) were affiliated with the *Chloroflexales* order, which belonged to the *Caldilineae* class, and these sequences were related to two genera of *Chloroflexus* and *Caldilinea*. The *Chloroflexus* genus sequences were seen in the four first and third cycle libraries, but did not occur in any second cycle libraries, and the *Caldilinea* genus sequences were only seen in three of the libraries representing the seedling stage of each cycle (Figure 5 and 6). Two OTUs (two clones) were affiliated with the *Thermomicrobiales* order, which belonged to the *Thermomicrobia* class, and were only seen in the two third cycle libraries (Figure 5). The *Kouleothrix* sequences (four OTUs) were seen in all the first and second cycle libraries, but not in any third cycle libraries. In contrast, the uncultured *Chloroflexi* sequences (five OTUs) were only seen in the four second and third cycle libraries (Figure 5).

The sequences related to the *Gemmatimonadetes* phylum accounted for 7.0% of both total clones, and valid OTUs. Twenty-two OTUs (94.9% of *Gemmatimonadetes* clones) were affiliated with the *Gemmatimonadetes* class and the other two OTUs were related to uncultured *Gemmatimonadetes* (Figure S10). All the sequences affiliated with the *Gemmatimonadetes* class were related to the *Gemmatimonadales* order and their abundance showed an increasing tendency during continuous cropping, accounting for 4.7, 6.2 and 8.7% respectively of the three cycle libraries (Figure 5). Clone diversity also showed a similar tendency. There were 9, 10 and 12 OTUs, respectively, in the three cycle libraries. The sequences related to uncultured *Gemmatimonadetes* were only seen in library M3 (Figure 6).

The sequences affiliated with the *Bacteroidetes*, *Planctomycetes* and *Verrucomicrobia* phyla were relatively abundant and diverse. These sequences accounted for 3.4, 3.5 and 4.4%, respectively, of the total clones and accounted for 4.7, 5.0, and 6.1%, respectively, of the total valid OTUs. Fourteen OTUs related to *Bacteroidetes* were affiliated with four classes: *Bacteroidia*, *Cytophagia*, *Flavobacteriia* and *Sphingobacteriia*, and were, respectively, related to the *Bacteroidales*, *Cytophagales*, *Flavobacteriales* and *Sphingobacteriales* orders (Figure S11). The remaining five OTUs were affiliated with uncultured *Bacteroidetes*. The analysis indicated that sequences related to the *Sphingobacteriales* order were only seen in the four first and second cycle libraries, but did not occur in any third cycle libraries (Figure 5). Sequences related to the *Bacteroidales* only were identified in the two second cycle libraries and sequences affiliated with *Flavobacteriales* only were seen in library S1. The other order or subdivision sequences didn’t show any significant tendencies over the three cycle libraries. All the sequences affiliated with the *Planctomycetes* phylum were related to the *Planctomycetales* order, which belonged to the *Planctomycetia* class (Figure S12). Clone abundance decreased in the second cycle libraries and increased significantly in the third cycle libraries, accounting for 3.1, 1.6 and 6.0%, respectively, of the clones identified in the three cycle libraries (Figure 5). Clone diversity showed a similar tendency and there were 5, 3 and 10 OTUs identified in the three cycle libraries, respectively. The sequences affiliated with the *Verrucomicrobia* phylum were related to the *Opitutae*, *Spartobacteria*, *Verrucomicrobiae* and uncultured *Verrucomicrobia* classes (Figure S13). The sequences related to the *Opitutae* and *Verrucomicrobiae* classes were affiliated with the *Opitutales* and *Verrucomicrobiales* orders, respectively. Sequences affiliated with the *Spartobacteria* class were not described. The analysis indicated that all orders or subdivisions affiliated with *Verrucomicrobia* did not show any significant dynamic change tendencies with continuous cropping.

## Discussion

In this study, 16S rRNA gene clone library analyses were undertaken to study soil bacterial succession under continuous peanut cultivation and warrant comparison with the previous study of soil eukaryotic microorganism succession [23]. The high diversity of soil bacteria in each growth stage library of the three peanut cropping cycles was shown based on the sequence analyses. A total of 343 OTUs were identified in the six libraries and these sequences were affiliated with 21 bacterial groups. Eight phyla: *Acidobacteria, Actinobacteria*, *Bacteroidetes*, *Chloroflexi*, *Gemmatimonadetes*, *Planctomycetes*, *Proteobacteria* and *Verrucomicrobia* were predominant, accounting for 93.0% of the 568 clones and 91.0% of the 343 valid OTUs. The bacterial communities identified in our libraries were much more diverse than the populations found by Sudini et al. [11] in a different peanut-cropping sequence soil, but the eight abundant bacterial groups identified in our libraries were also all identified by Sudini et al. [11] and the *Proteobacteria* and *Acidobacteria* phyla were common predominant members. There have been few reports about specific soil bacterial phylotype succession with continuous cropping of plants, but, taking into account previous reports on the root associated soil microbial communities around clover, bean or alfalfa [12], there is a suggestion that plant species could have a relatively greater impact than soil type and plant age in determining the soil microbial community structure. Consequently, the analyses of bacterial succession in this study can provide general information about the relationships between the soil microbial community and continuous peanut cropping.

It is known that soil bacterial populations are influenced by a wide range of factors. Soil type, plant species and cropping patterns are the factors that most affect the bacterial community structure in soil [6], [11], [12]. In order to reduce interference from other factors and truly reflect the relationship between soil bacterial succession and continuous cropping with peanut, several measures were used, as described in the Materials and Methods. Cluster analyses demonstrated that the soil bacterial assemblages obtained from the same cropping cycle were similar, regardless of growth stage. Heatmap analyses and phylogenetic analyses also showed dynamic changes in bacterial populations with continuous peanut cropping. This was consistent with the earlier study into soil eukaryotic microorganisms [23]. These results demonstrated that the soil microbial communities were indeed affected by continuous cropping of peanut. It has been reported that soil microbial biomass and structure were also significantly influenced by continuous cropping with cucumber, maize, rice and pea [21], [22], [32], [33]. These findings indicated that successional change in soil microbial communities with continuous cropping could be a common feature.

Analyses of sequences obtained in the six libraries showed that the successional trends were complex and can be mainly divided into four types: trends showing a continuous increase or decrease; a trend where the population increases and then decreases or a trend where the population deceases and then increases.

Data analyses showed that the abundance and/or diversity of clones affiliated with *Acidobacteriales*, *Chromatiales*, *Gemmatimonadales*, *Myxococcales* and *Sphingomonadales* were relatively abundant in the libraries and all successively increased with continuous peanut cropping. In addition, some populations that were not abundant in the libraries, including the *Caulobacterales*, *Desulfuromonadales*, *Gaiellales*, *Thermomicrobiales* phylotypes and two uncultured bacterial phylotypes: uncultured *Chloroflexi* and uncultured *Gemmatimonadetes*, also showed an successively increasing tendency with continuous peanut cropping.

Other phylotypes showed the opposite tendency with continuous peanut cropping. Their abundance and/or diversity successively decreased during continuous cropping. These phylotypes, including: *Actinomycetales*, *Burkholderiales* and *Pseudomonadales,* were relatively abundant in the libraries. Additionally, many non-abundant phylotypes identified in our libraries, including: *Alteromonadales*, *Methylophilales, Rhodocyclales*, *Sphingobacteriales* and two uncultured phylotypes (uncultured *Acidobacteria* and uncultured *Actinobacteridae*) also showed a successively decreasing tendency with continuous cropping.

There were another two different dynamic tendencies during continuous peanut cropping. The abundance and/or diversity of *Anaerolineales* and *Kouleothrix* increased with continuous cropping in the first and second cycles but disappeared in the third cycle libraries. The phylotypes of *Bacteroidales*, *Legionellales* and *Oceanospirillales* appeared in the second cycle libraries, but not occurred in any of the first and third cycle libraries. All these phylotypes were not abundant in the libraries. In contrast, the abundance and/or diversity of some phylotypes decreased with continuous cropping in the first and second cycles but increased in the third cycle libraries. These phylotypes were *Planctomycetales*, *Rhizobiales*, *Rhodospirillales*, *Xanthomonadales* and the *Chloroflexus* genus which belonged to *Chloroflexales*.

The complex dynamics of the bacterial populations identified in this study raises many questions, such as why did these populations change over time and what roles did the changing bacterial population play during continuous cropping?

Bacterial populations in soil that are affiliated with the *Alteromonadales*, *Burkholderiales, Flavobacteriales*, *Pseudomonadales, Rhizobiales* and *Rhodospirillales* orders have been reported as being beneficial to plant growth [3], [4], [34], [35]. These populations promoted plant growth by being involved in nitrogen fixation, phosphorus solubilization and in the biocontrol of plant pathogens [4], [34]. Interestingly, the abundance and/or diversity of these populations identified in our libraries all showed a decreasing tendency with continuous cropping. Most of these populations showed a successively decreasing tendency and the populations related to *Rhizobiales* and *Rhodospirillales* also showed a decreasing tendency in the first two continuous cropping cycles, but increased in the third cycle libraries. It has been reported that bacteria can quickly respond to environmental changes [36] and PGPR (plant growth promoting rhizobacteria) can have positive effects on plant vigor and productivity, especially under stress conditions [34], [37], [38]. So the populations associated with *Rhizobiales* and *Rhodospirillales* could be quick responders under continuous cropping stress. Based on these analyses, it can be seen that the beneficial bacterial population showed a simplified tendency with continuous cropping, but the succession models were non-single.

The *Pseudomonales* are considered a major group of rhizobacteria that may have the potential to biologically control plant pathogens [34], [39]. The abundance and diversity of the sequences of this phylotype identified in the libraries decreased successively with continuous peanut cropping. It was reported that *Fusarium* genus was major pathogens of plant root rot, and *Pseudomonas* was major antagonists [40], [41], [42]. Interestingly, contrary to the decrease tendency of *Pseudomonales,* sequences affiliated with *Fusarium* showed increasing tendency with continuous cropping of peanut [23], [43]. The succession analyses of soil eukaryotic microorganisms in the earlier study demonstrated that the pathogenic and beneficial fungi were positively selected over time and showed, respectively, increased and simplified tendencies with continuous cropping [23]. These analyses, along with previous studies [26], [33], [44], demonstrated that the trend towards an increase in microbial pathogens and simplification of the beneficial microbial community could be important factors contributing to the decline in peanut growth and yield over many years of continuous cropping.

Plant–microbial interactions in soil are the determinants of plant health and soil fertility. However, due to the high diversity and heterogeneity of soil bacteria, the interactions between bacteria, soil and plants, especially for specific bacteria, are not well understood. Many of the bacterial phylotypes identified in our libraries showed dynamic change during continuous cropping, but only a few have been reported as being able to promote plant growth and the functions of the other phylotypes have not been identified yet. Generally, PGPRs function in the following ways: they synthesize phytohormones, fix nitrogen, solubilize phosphorus, facilitate the uptake of certain nutrients from soil, increase plant tolerance to extreme environmental conditions and lessen or prevent plant diseases [4], [34], [35]. In contrast, other rhizobacteria suppress plant growth by acting as a carbon drain and causing disease, galls and tumors on plants [45], [46]. These reports, combined with the bacterial succession dynamic analyses in this study, could provide the basis for further study into the interactions between soil bacteria and continuous peanut cropping and the decline in peanut growth and yield under continuous cropping.

In addition, the analyses indicated that some bacterial populations also were affected by the growth stage of peanut. The abundance and diversity of sequences affiliated with the *Acidobacteriales* order in the pod-maturing stage library were higher than in the seedling stage library of the same cropping cycle. Oppositely, the clone abundance and diversity affiliated with *Burkholderiales* and uncultured γ-*proteobacteria* in the seedling stage libraries were higher than in the pod-maturing stage libraries of the same cycle. In addition, sequences affiliated with *Bdellovibrionales* and the *Caldilinea* genus, which belong to *Chloroflexales*, *Solirubrobacterales*, and uncultured δ*-proteobacteria* were only seen in the seedling stage libraries of the three cycles, but did not occur in any pod-maturing stage libraries. Based on the analyses, it can be seen that these populations may be related to the growth and development of peanut. It was reported that the rhizobacterial communities were minor affected by plant development [12], [47]. Interestingly, the eukaryotic microorganisms did not show any obvious correlation with peanut development in the earlier study [23]. There needs to be further study into the interaction mechanisms between microbial communities and the growth or development of peanut.

In this study and in a previous study [23], the succession dynamics of soil microbial communities with continuous peanut cropping has been comprehensively analyzed. Future studies will investigate the interactions between the microbial populations that showed a change over time and the mechanisms behind the interactions between microbes and peanut under continuous cropping.

## Supporting Information

Figure S1 **Rarefaction curves for the 16S rRNA gene libraries constructed from each of the soil samples.**
(TIF)

Figure S2 **Cluster membership based on a k-means clustering analysis of the 16S rRNA gene clone libraries** (Case number 1–6 were library S1, M1, S2, M2, S3 and M3, respectively).
(TIF)

Figure S3 **Phylogenetic tree representing affiliations of the 16S rRNA gene sequences related to the α** ***-proteobacteria*** **class.**
(TIF)

Figure S4 **Phylogenetic tree representing affiliations of the 16S rRNA gene sequences related to the γ** ***-proteobacteria*** **class.**
(TIF)

Figure S5 **Phylogenetic tree representing affiliations of the 16S rRNA gene sequences related to the β** ***-proteobacteria*** **class.**
(TIF)

Figure S6 **Phylogenetic tree representing affiliations of the 16S rRNA gene sequences related to the δ** ***-proteobacteria*** **class.**
(TIF)

Figure S7 **Phylogenetic tree representing affiliations of the 16S rRNA gene sequences related to the** ***Acidobacteria*** **phylum.**
(TIF)

Figure S8 **Phylogenetic tree representing affiliations of the 16S rRNA gene sequences related to the** ***Actinobacteria*** **phylum.**
(TIF)

Figure S9 **Phylogenetic tree representing affiliations of the 16S rRNA gene sequences related to the** ***Chloroflexi*** **phylum.**
(TIF)

Figure S10 **Phylogenetic tree representing affiliations of the 16S rRNA gene sequences related to the** ***Gemmatimonadetes*** **phylum.**
(TIF)

Figure S11 **Phylogenetic tree representing affiliations of the 16S rRNA gene sequences related to the** ***Bacteroidetes*** **phylum.**
(TIF)

Figure S12 **Phylogenetic tree representing affiliations of the 16S rRNA gene sequences related to the** ***Planctomycetes*** **phylum.**
(TIF)

Figure S13 **Phylogenetic tree representing affiliations of the 16S rRNA gene sequences related to the** ***Verrucomicrobia*** **phylum.**
(TIF)
