# Supplementary figures and images for: Dynamic Succession of Soil Bacterial Community during Continuous Cropping of Peanut (Arachis hypogaea L.)
Source: PLoS One. 2014 Jul 10;9(7):e101355. doi: 10.1371/journal.pone.0101355 (PMC4092034; doi:10.1371/journal.pone.0101355)

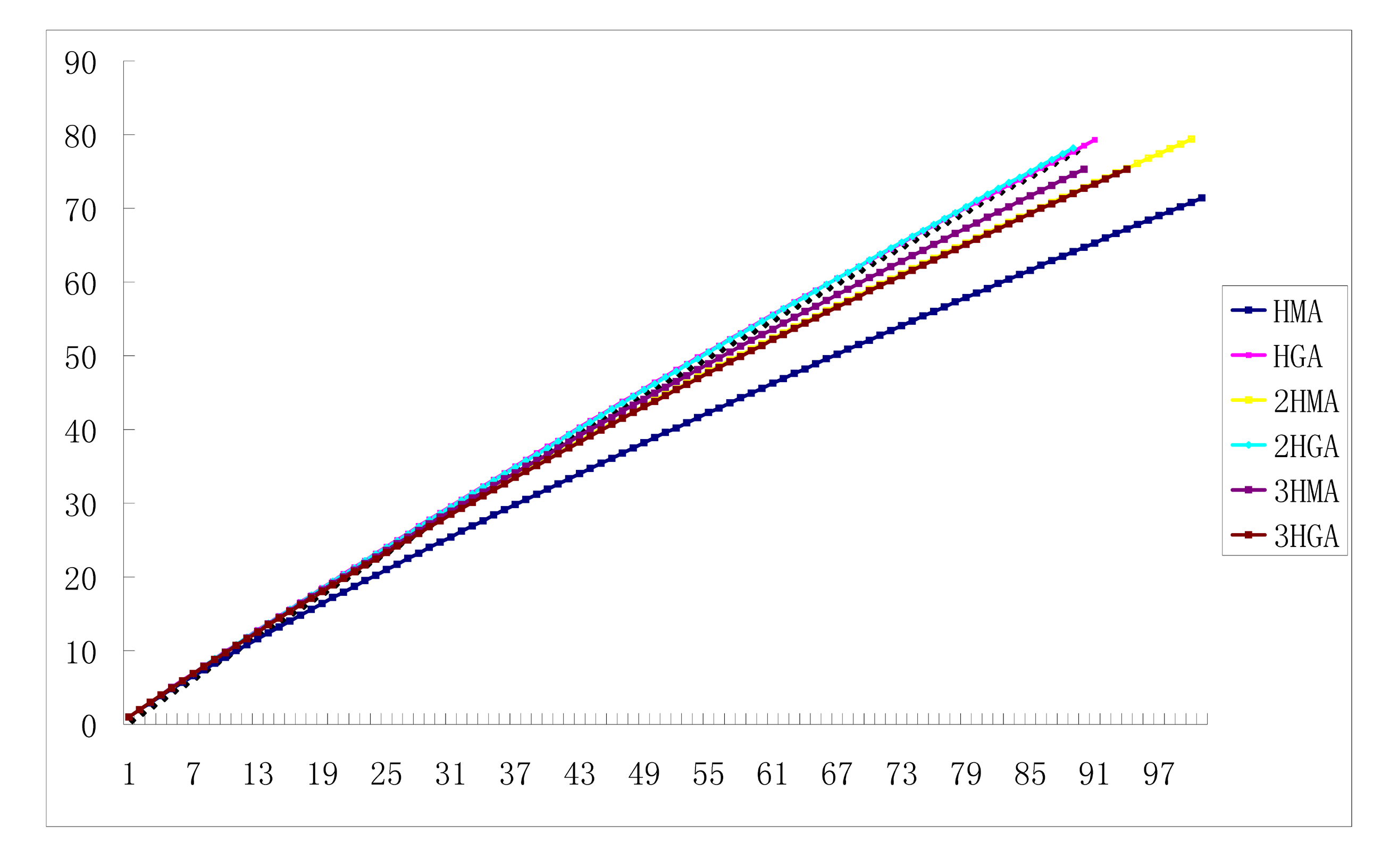

Supplement: Figure S1 — Rarefaction curves for the 16S rRNA gene libraries constructed from each of the soil samples. (TIF) [file pone.0101355.s001.tif]

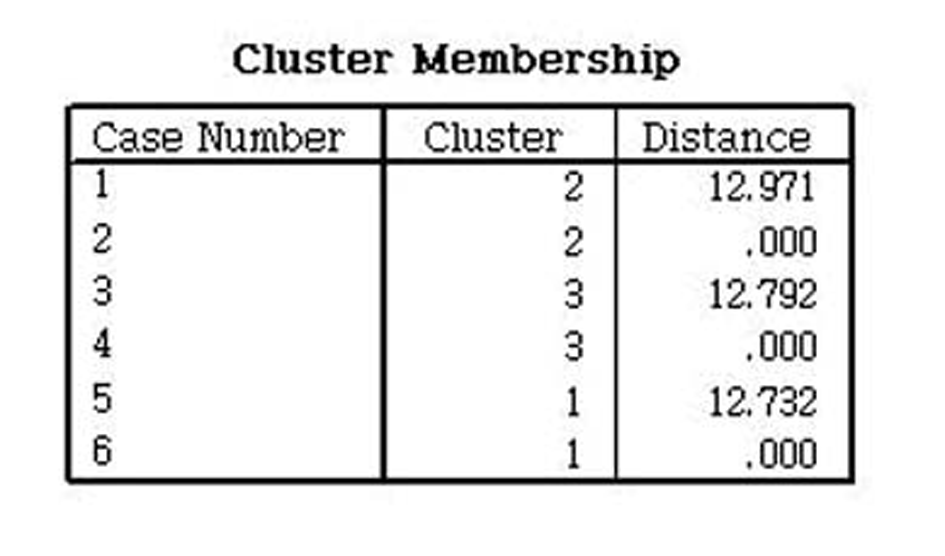

Supplement: Figure S2 — Cluster membership based on a k-means clustering analysis of the 16S rRNA gene clone libraries (Case number 1–6 were library S1, M1, S2, M2, S3 and M3, respectively). (TIF) [file pone.0101355.s002.tif]

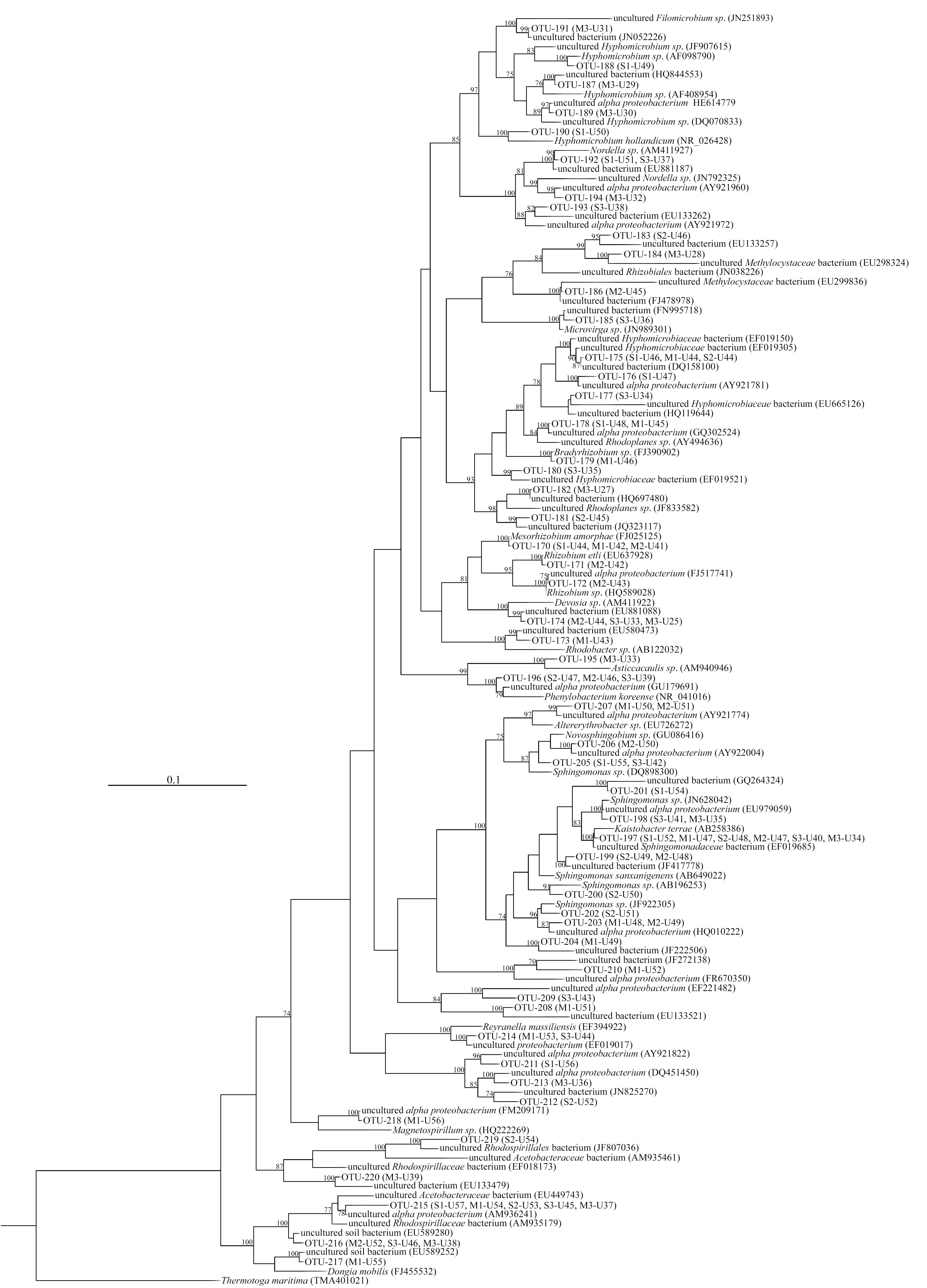

Supplement: Figure S3 — Phylogenetic tree representing affiliations of the 16S rRNA gene sequences related to the α -proteobacteria class. (TIF) [file pone.0101355.s003.tif]

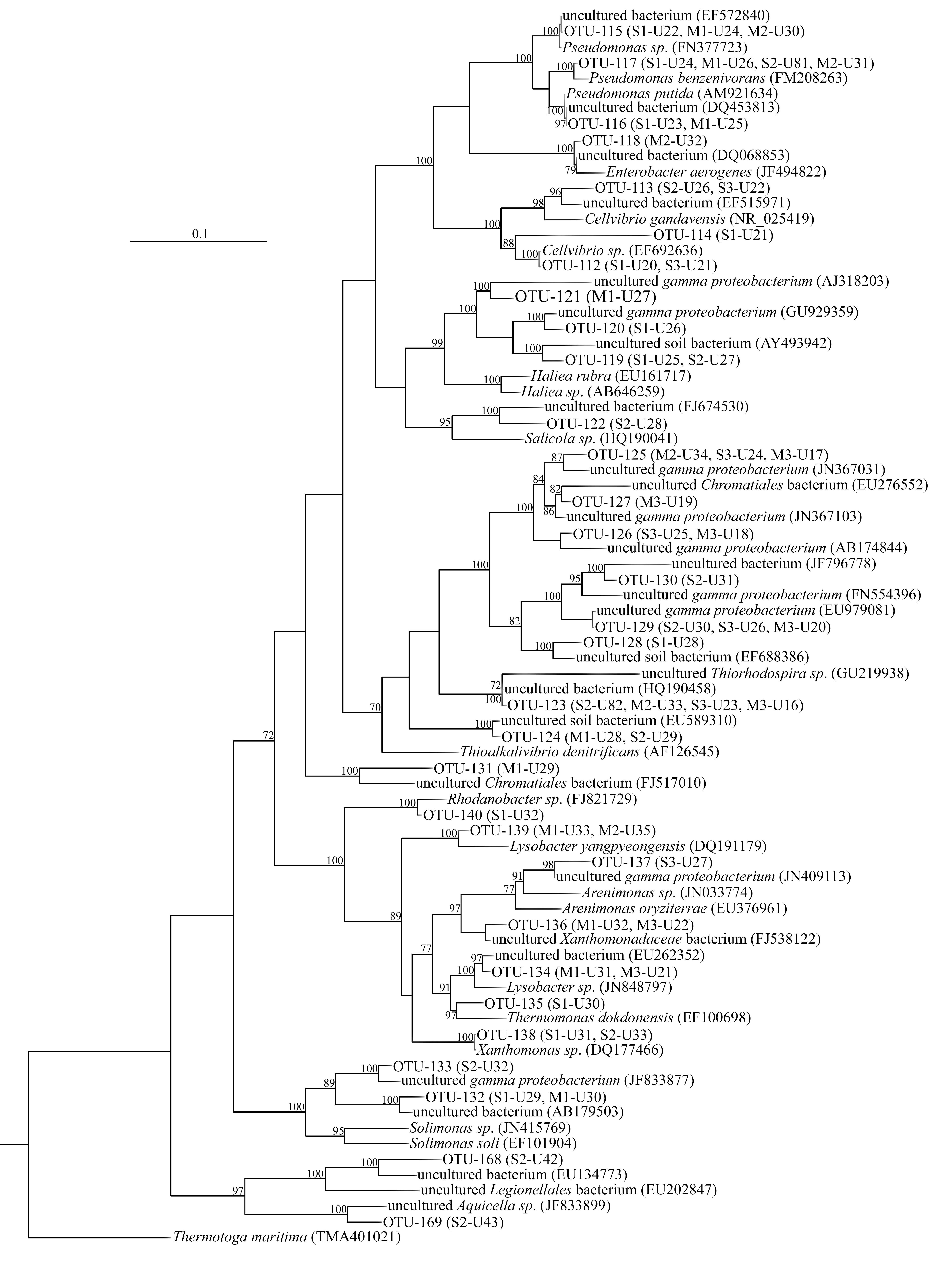

Supplement: Figure S4 — Phylogenetic tree representing affiliations of the 16S rRNA gene sequences related to the γ -proteobacteria class. (TIF) [file pone.0101355.s004.tif]

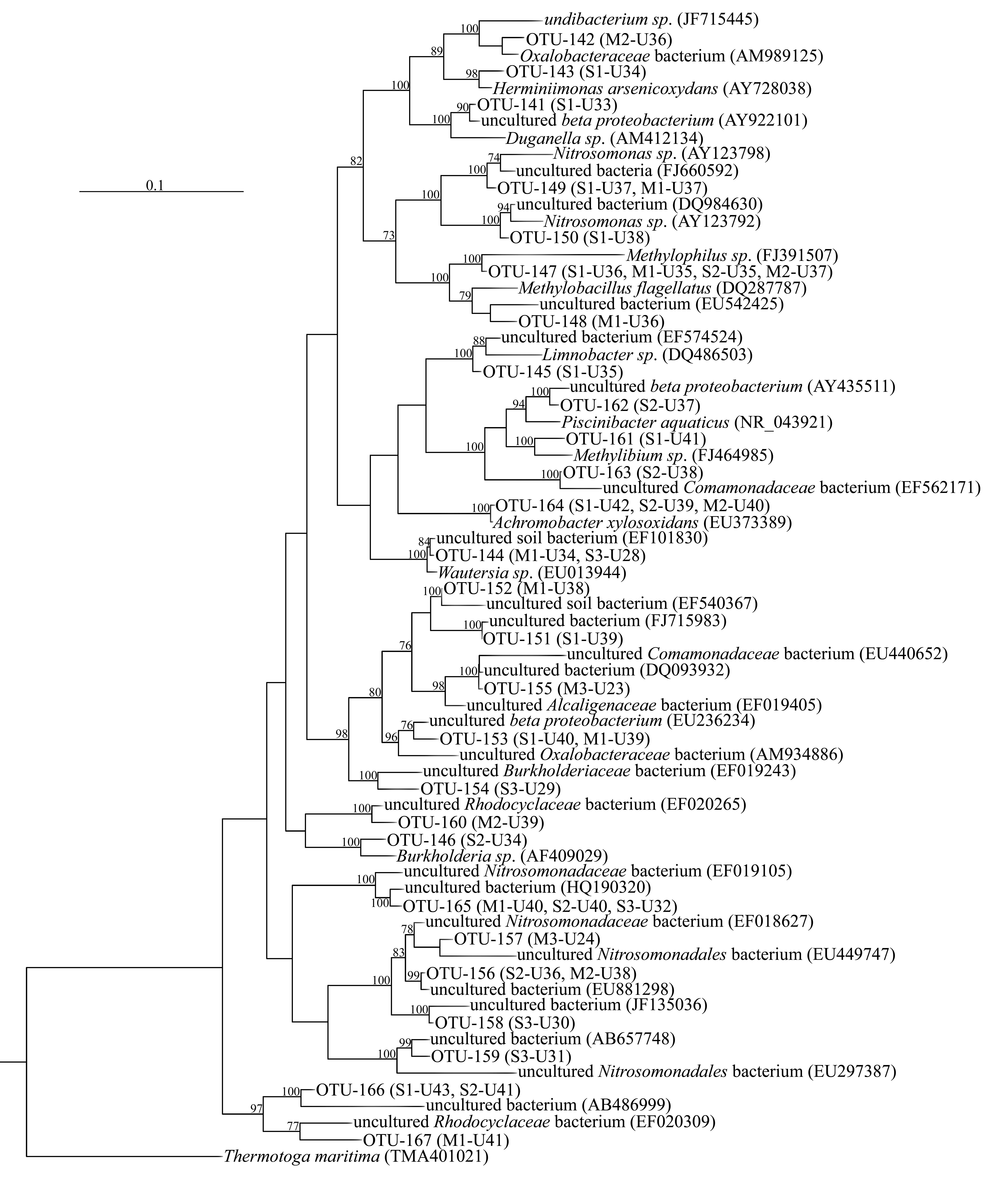

Supplement: Figure S5 — Phylogenetic tree representing affiliations of the 16S rRNA gene sequences related to the β -proteobacteria class. (TIF) [file pone.0101355.s005.tif]

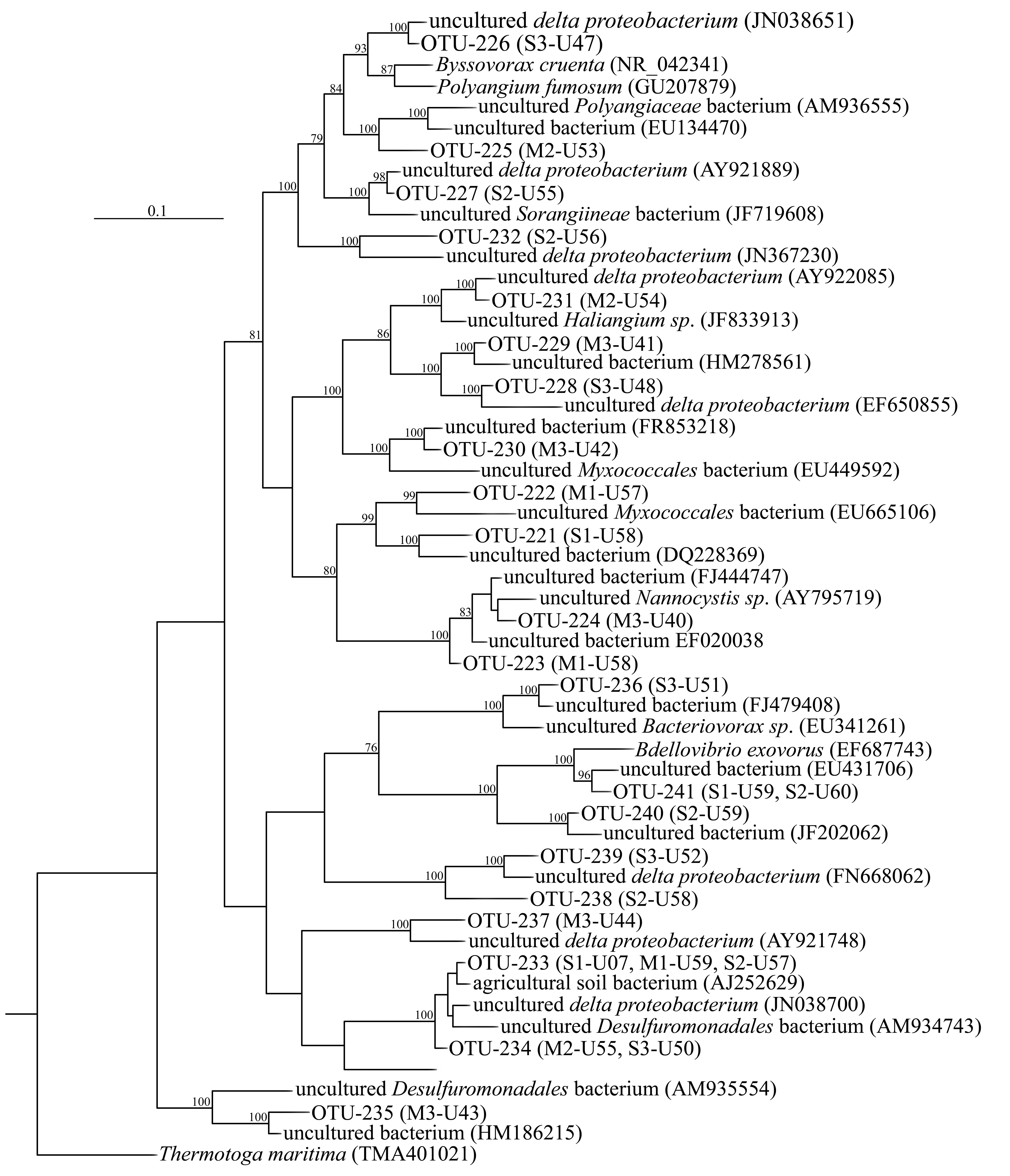

Supplement: Figure S6 — Phylogenetic tree representing affiliations of the 16S rRNA gene sequences related to the δ -proteobacteria class. (TIF) [file pone.0101355.s006.tif]

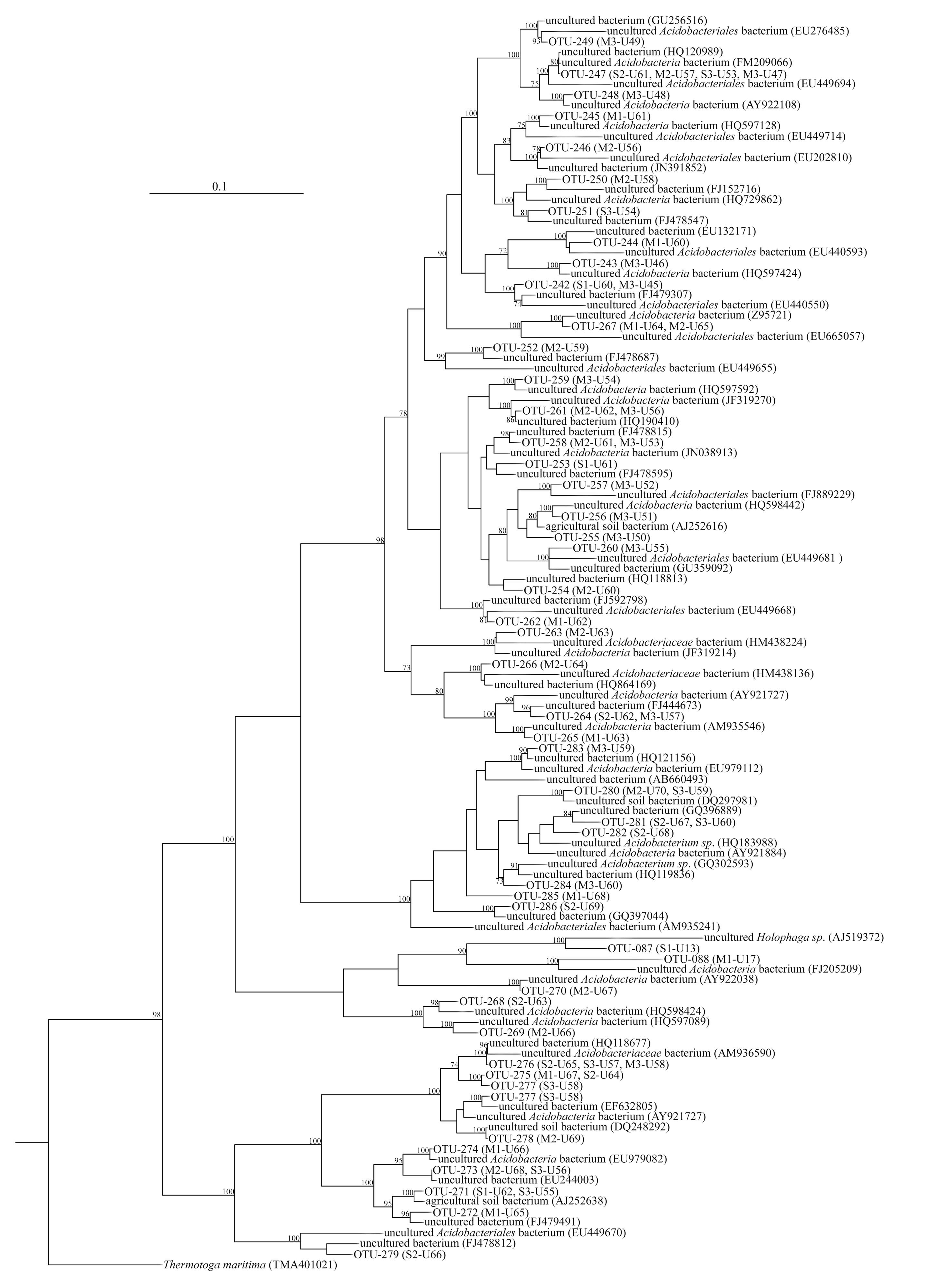

Supplement: Figure S7 — Phylogenetic tree representing affiliations of the 16S rRNA gene sequences related to the Acidobacteria phylum. (TIF) [file pone.0101355.s007.tif]

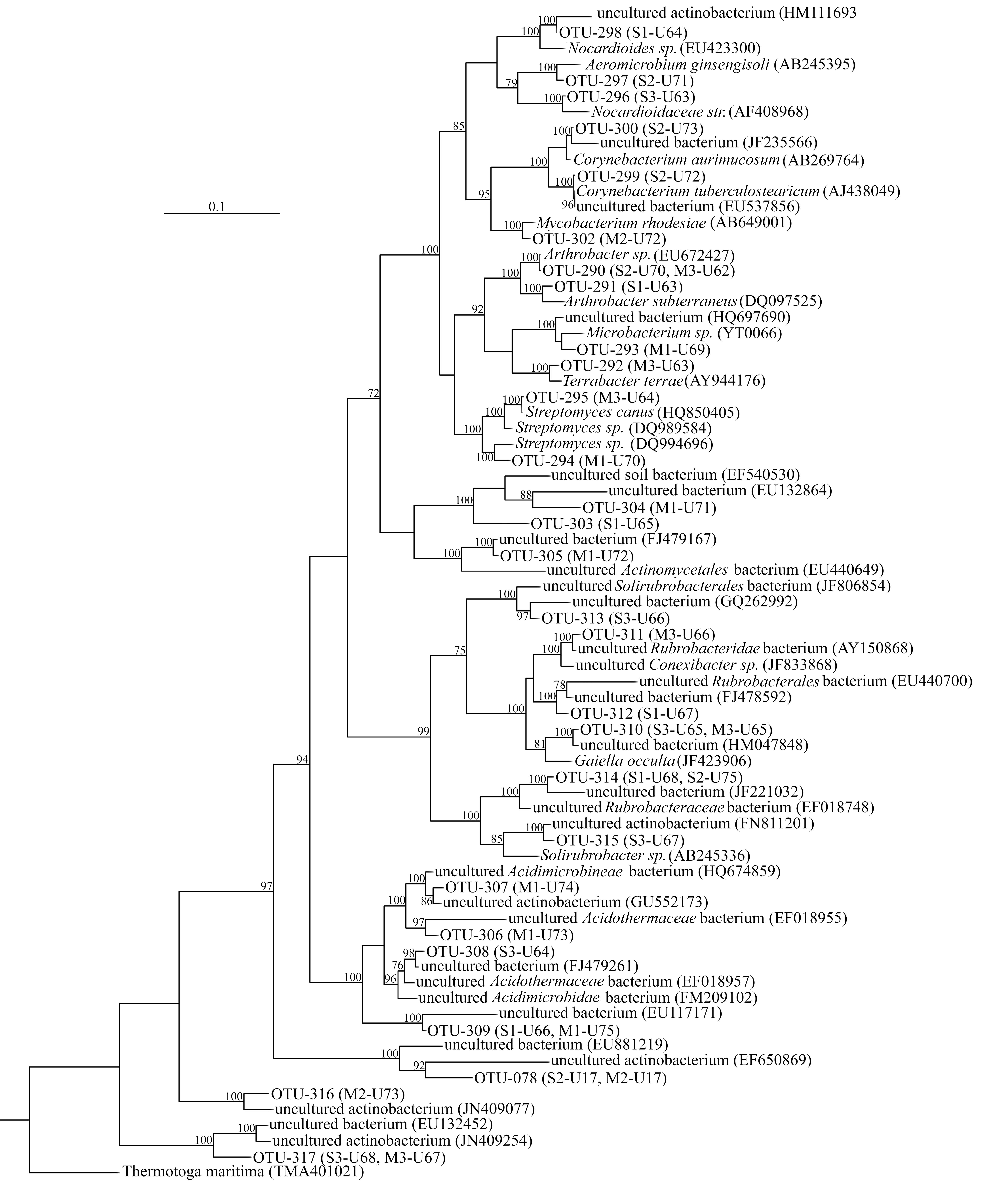

Supplement: Figure S8 — Phylogenetic tree representing affiliations of the 16S rRNA gene sequences related to the Actinobacteria phylum. (TIF) [file pone.0101355.s008.tif]

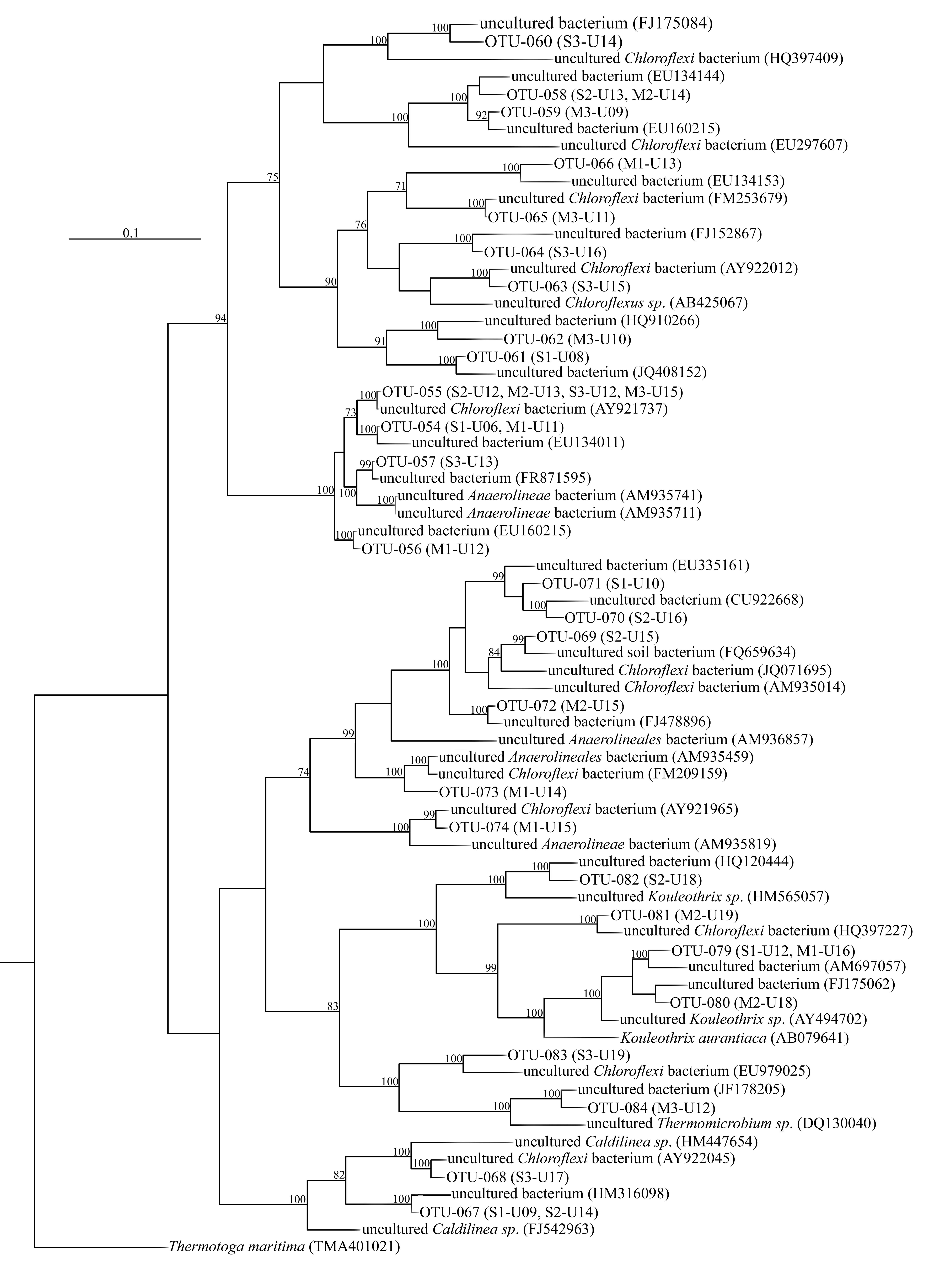

Supplement: Figure S9 — Phylogenetic tree representing affiliations of the 16S rRNA gene sequences related to the Chloroflexi phylum. (TIF) [file pone.0101355.s009.tif]

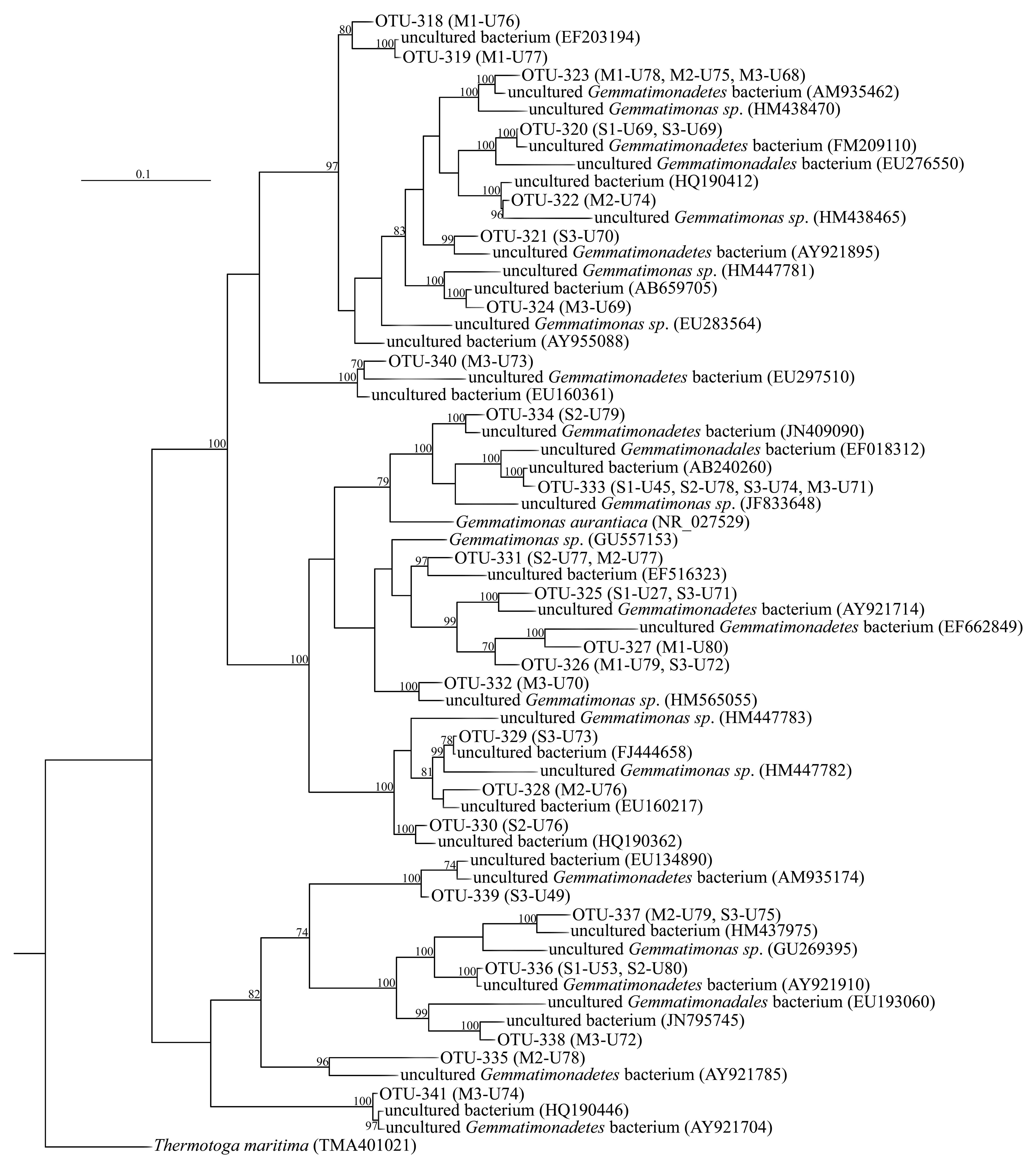

Supplement: Figure S10 — Phylogenetic tree representing affiliations of the 16S rRNA gene sequences related to the Gemmatimonadetes phylum. (TIF) [file pone.0101355.s010.tif]

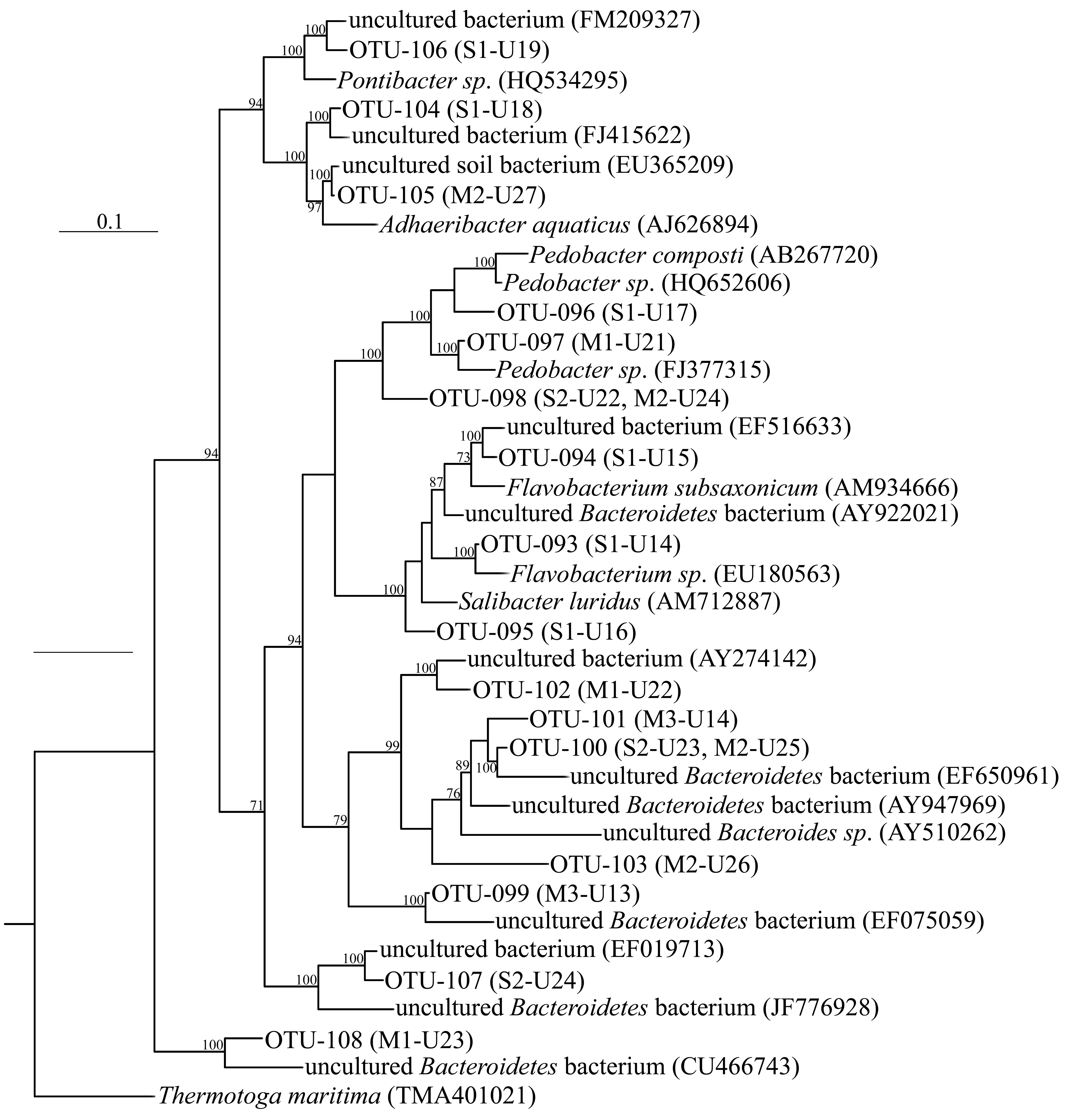

Supplement: Figure S11 — Phylogenetic tree representing affiliations of the 16S rRNA gene sequences related to the Bacteroidetes phylum. (TIF) [file pone.0101355.s011.tif]

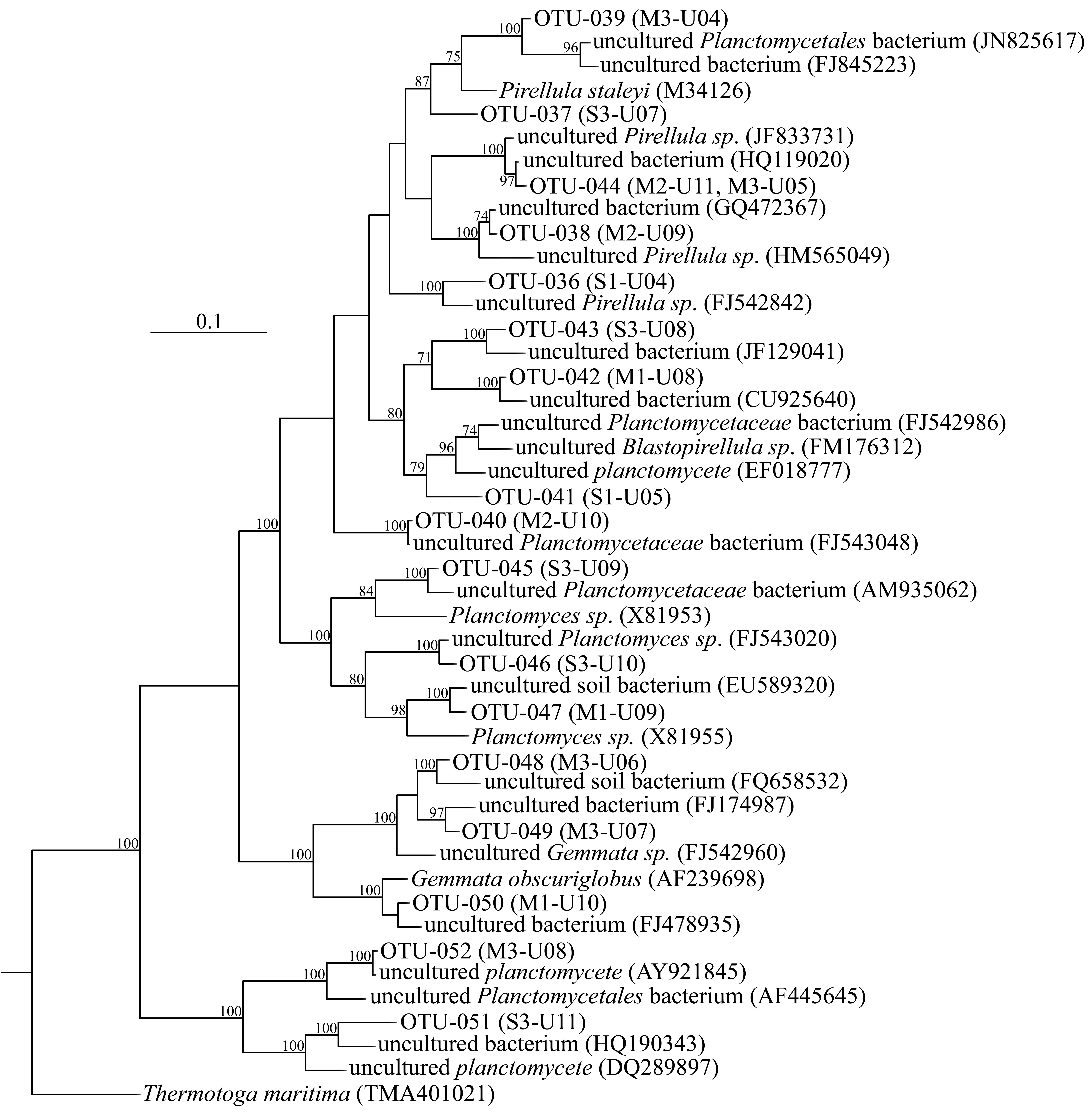

Supplement: Figure S12 — Phylogenetic tree representing affiliations of the 16S rRNA gene sequences related to the Planctomycetes phylum. (TIF) [file pone.0101355.s012.tif]

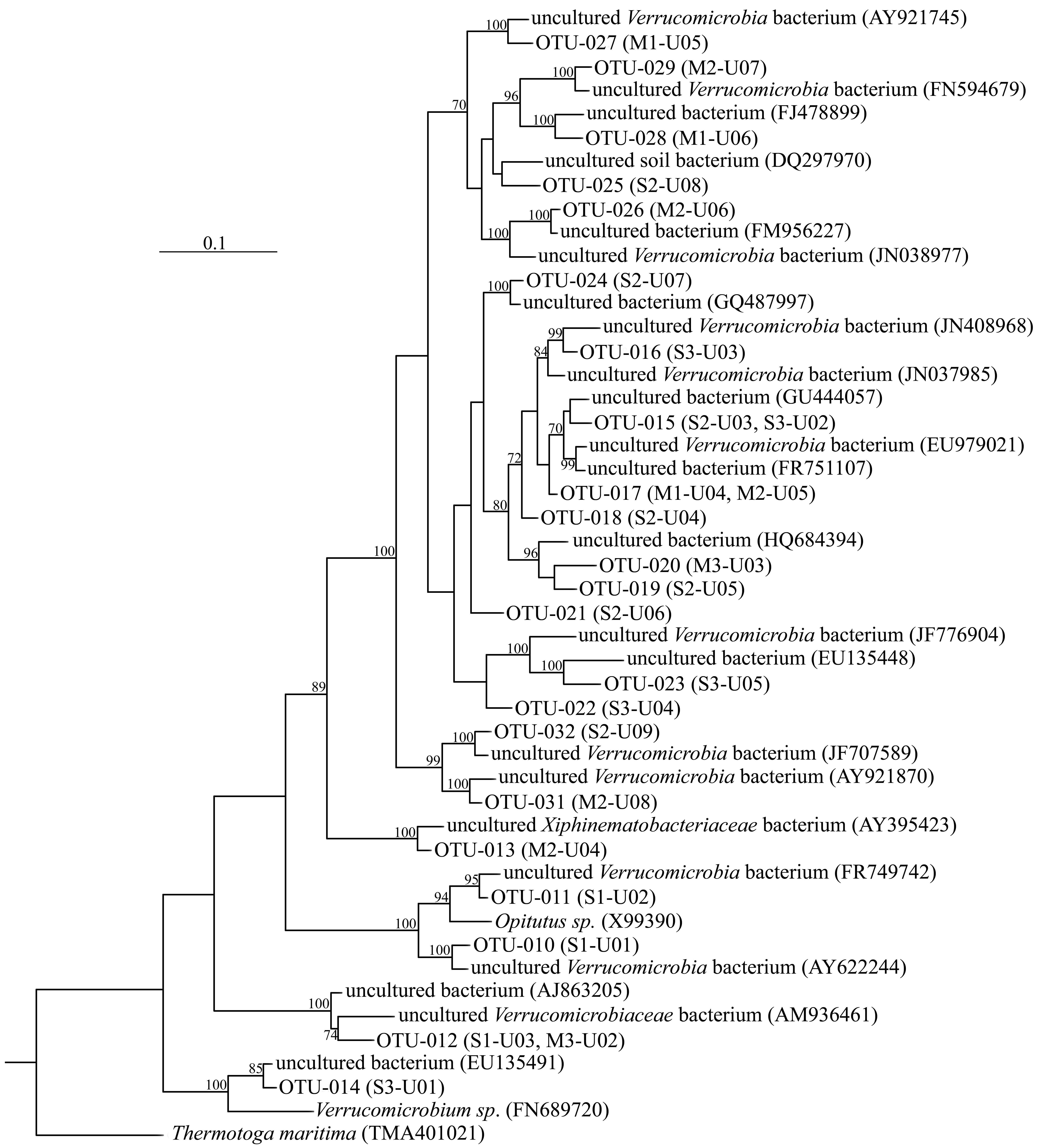

Supplement: Figure S13 — Phylogenetic tree representing affiliations of the 16S rRNA gene sequences related to the Verrucomicrobia phylum. (TIF) [file pone.0101355.s013.tif]
